# Use of herbal medicinal products among children and adolescents in Germany

**DOI:** 10.1186/1472-6882-14-218

**Published:** 2014-07-02

**Authors:** Yong Du, Ingrid-Katharina Wolf, Wanli Zhuang, Stefanie Bodemann, Werner Knöss, Hildtraud Knopf

**Affiliations:** 1 Department of Epidemiology and Health Monitoring, Robert Koch Institute, General-Pape-Str. 62-66, Berlin 12101, Germany; 2 Complementary and Alternative Medicines - Traditional Medicinal Products, Federal Institute for Drugs and Medical Devices, Kurt-Georg-Kiesinger-Allee 3, Bonn 53175, Germany

**Keywords:** Herbal medicinal products, Children, KiGGS, Germany

## Abstract

**Background:**

Germany is a country with a high use of herbal medicinal products. Population-based data on the use of herbal medicinal products among children are lacking. The aim of this study is to investigate the prevalence, patterns and determinants of herbal medicine use among children and adolescents in Germany.

**Methods:**

As data base served the German Health Interview and Examination Survey for Children and Adolescents (KiGGS), a representative population based survey conducted 2003–2006 by the Robert Koch Institute. 17,450 boys and girls aged 0–17 years provided information on drug use in the preceding seven days. Herbal medicinal products were defined according to the European and German drug laws. SPSS Complex Sample method was used to estimate prevalence rates and factors associated with herbal medicine use.

**Results:**

The prevalence rate of herbal medicinal product use amounts to 5.8% (95% confidence interval 5.3-6.3%). Use of herbal medicine declines along with increasing age and shows no difference between boys and girls in younger age groups. Teenage girls are more likely to use herbal medicines than teenage boys. Two thirds of herbal medicines are used for the treatment of coughs and colds; nearly half of herbal medicines are prescribed by medical doctors. Determinants of herbal medicinal product use are younger age, residing in South Germany, having a poor health status, having no immigration background and coming from a higher social class family. Children’s and parents-related health behavior is not found to be associated with herbal medicine use after adjusting for social class.

**Conclusions:**

Use of herbal medicinal products among children and adolescents between the ages of 0 and 17 years in Germany is widely spread and shows relatively higher rates compared to international data. This study provides a reference on the use of herbal medicinal products for policy-makers, health professionals and parents. Further studies are needed to investigate the effectiveness and safety of specific herbal medicinal products, potential effects of long term use as well as possible interactions of herbal medicinal products with concomitantly used conventional medicines.

## Background

The use of herbal medicinal products for the treatment and prevention of diseases has a long tradition worldwide. Nowadays, it still plays an important role in the health care of numerous divergent societies ranging from developing countries in Asia and Africa to western developed nations [1]. In countries with highly developed health care systems, herbal medicine is often regarded as a complementary and alternative medicine (CAM), thus less common in clinical settings, but has become increasingly popular in recent years [2-4]. Specific provisions for herbal medicinal products within the European and German legal framework dealing with health care reflect its recognized significance. In this context, herbal medicinal products are defined as medicinal products containing exclusively herbal active substances [5,6]. These differ from chemically defined substances in several aspects; most notably, they consist of complex multi-component mixtures resulting from, for example, extraction of plant parts such as roots and leaves. Despite their wide use, many frequently used herbal medicinal products have not undergone complex scientific analysis via clinical trials regarding safety and efficacy [7,8]. One of the reasons for this is the fact, that for the majority of herbal active substances, therapeutic activity cannot be related to scientifically identified, chemically defined ingredients, which aggravates conventional clinical studies. Taking into consideration these particularities of herbal medicinal products, European legislation as well as the German Medicines Act specifically address resulting characteristics [5,6] and accommodate adapted rules for marketing authorizations of herbal medicinal products.

Concerning the use of medicinal products in children and adolescents, it is generally recognized that preclinical and clinical studies in this field are lacking regardless of the products’ classification as herbal or chemically defined products [9]. Additionally, many medicinal products, particularly herbal medicines, are used off-label in children [10]. Even though herbal medicinal products are frequently used and regarded as ‘natural’ products, they can also cause adverse drug reactions [11,12] as well as adverse interactions with other medications [13,14].

Pediatric use of herbal medicinal products has been investigated in previous studies mainly under the umbrella of CAM. However, a differentiation according to the type of CAM (homoeopathy, herbal medicine, manual therapies, etc.) is necessary as various types of CAM exhibit diverse characteristics and may have a different effect on the users’ health. Additionally, users’ profiles could vary according to type of CAM [15].

Most of the studies are conducted among children with a specific chronic condition [16-19] or among inpatients and outpatients [20-23]. Internationally, only a few population-representative studies investigate herbal medicinal product use among children in the general population [24-26]. The prevalence of herbal medicinal product use in these studies is rather low, ranging from less than 0.5% in the last 7 days among children 0–12 years [25], and 3.9% in the last 12 months among children 0–17 years in the USA [26], to 2.4% in the past 3 years among children 0–13 years in Italy [24]. Further differentiated analysis of patterns and determinants of herbal medicinal product use is impossible, because of small numbers of users in these studies [24-26].

Traditionally, Germany is a country with a high use of herbal medicinal products. A German study conducted in two birth cohorts [15] and other German studies among pediatric outpatients with small sample sizes [20,27,28], support this, but there are no representative epidemiological studies investigating herbal medicinal product use in the general child population. The present study attempts to fill this knowledge gap. Using the representative data from the latest German Health Interview and Examination Survey for Children and Adolescents (KiGGS), we present here the prevalence rates, patterns and determinants of herbal medicinal product use among non-institutionalized children and adolescents in Germany.

## Methods

### Data source and study population

The German Health Interview and Examination Survey for Children and Adolescents (KiGGS) was conducted by the Robert Koch Institute between May 2003 and May 2006. In this survey, a highly standardized protocol encompassing a personal medical computer-assisted interview administrated by physicians (including the drug use interview), self-administered questionnaires, and standardized physical examinations were conducted [29]. The design, sampling strategy and study protocol have been described in detail elsewhere [29]. Briefly, survey participants were enrolled by a two-stage sampling procedure. In the first stage, a sample of 167 municipalities was drawn which were representative of municipality sizes and structures in Germany. Stratified by sex and age, random samples of children and adolescents between the ages of 0 and 17 years were then drawn from local population registries in proportion to the age and sex structure of Germany’s child population. A response rate of 66.6% resulted in a final sample of 17,641 children and adolescents. 191 study participants did not take part in the drug use interview and were excluded, resulting in a basic population of 17,450 (8,880 boys, 8,570 girls) available for final analysis.

Non-response analysis showed little variation between the age groups and sexes, and no difference was found with respect to health-related variables [29,30].

The survey was approved by federal data protection officials and by the Charité Universitätsmedizin Berlin medical ethics committee (authorization number: 101/2000). Written informed consent was obtained prior to the interview and examination from the children’s parents and the children themselves if they were aged 14 years or older. Authors have full access to KiGGS data for this study.

### Data collection

As described elsewhere in detail [29], standardized, age-specified (0–2, 3–6, 7–10, 11–13 and 14–17 years) self-filled questionnaires were completed by parents (over 80% of questionnaires were completed by children’s mothers). Parallel self-filled questionnaires were filled out by adolescents (11–13 and 14–17 years), including for example questions on sports activities. Data collected comprised socio-demographic characteristics, family economic background as well as children’s and parents-related health behavior.

Drug use data were collected in a computer-assisted standardized personal interview conducted by a physician by the following question:

*Has your child taken any medicines in the last seven days? Please also mention the use of any ointments, liniments, contraceptive pills, vitamin and mineral supplements, medicinal teas, herbal medicinal products and homoeopathic medicinal products*.

To facilitate the investigation and verification of drug use, parents were asked in advance to bring prescriptions or original packages to the examination sites.

In the computer-assisted standardized personal interview, children aged 14 years and older were encouraged to supplement data on the use of medicines themselves. Details of medication use were documented, such as brand name, condition(s) treated (as many as two conditions could be provided and recorded), origin (prescribed either by a medical docotr, or a non-medical practition, bought over the counter, or obtained from other sources), duration of use (<1 week, 1–4 weeks, 1–12 months or 1 year or longer), self-rated improvement of condition(s) treated (greatly, partly, not much, or not at all), as well as any adverse drug events following the intake of the medicine. Specific ATC (Anatomical Therapeutic Chemical) codes were assigned to all reported medications, and WHO ICD-10 codes to the conditions for which the medications were taken [31].

### Identifying herbal medicinal products from drug database

Of 17,450 study participants, 8,899 were users of medicine. They utilized a total of 14,588 preparations within the last 7 days. Each preparation was primarily classified by health care professionals according to the comprising active substance(s) and categorized as ‘conventional medicinal product’ , ‘herbal medicinal product’ , ‘homoeopathic medicinal product’ , ‘combination product’ , or ‘not attributable’. This process of classification was systematically conducted by consulting at first the German drug dictionary “Gelbe Liste 2005” [32]. If this information was insufficient, the “Drug Information System (AMIS)” of the German Institute of Medical Documentation and Information (DIMDI) [33] and then “Rote Liste 2005” [34], another German drug dictionary, were consulted. If the information was still insufficient an internet research was conducted and if this was also unsuccessful the product was classified as ‘not attributable’.

For the present study, apart from the group of ‘conventional medicinal product’ with clearly chemically defined active substances, all other preparations were further combed one by one by health care professionals to control for correct classification into the specific subgroups. In a second round, experts from BfArM (Federal Institute for Drugs and Medical Devices, Germany) independently reassessed all these drug classifications. Bearing in mind their regulatory background, those experts assigned all herbal medicinal products according to the definition of Directive 2001/83/EC [5], respectively, the German Medicines Act [6]. Therefore, the final data for analysis exclusively included those products which could be identified, regardless of the dosage form, to comply with the legal definitions for ‘herbal medicinal products’ or ‘traditional herbal medicinal products’ as given in current European and German drug law. Other products such as combination products (containing both chemically defined and herbal active substances), cosmetics, food products or medical devices or which could not be assigned – were excluded from the analysis. The classification processes came to the result that 10,433 preparations belonged to the group ‘conventional medicinal product’ and 1,152 were identified as herbal medicinal products.

### Definition of co-variables

We included several co-variables in the analysis that are likely to be associated with children’s herbal medicinal product use. These co-variables cover children’s demographics (e.g. age, sex, residential region, immigration background [35]), children’s health status and health behavior as well as parental socio-economic status and health behavior.

### -Children’s health status and health behavior

Children’s general health status was rated by their parents with the question ‘How do you rate the health status of your child at large?’ The answer choices were ‘excellent/very good’ , ‘good’ , ‘fair’ , ‘bad’ and ‘very bad’. Further, we used the Children with Special Health Care Needs (CSHCN) screener tool to identify children with special health care needs [36].

Children’s body mass index (BMI) was computed based on measurement of children’s weight and height. Relative body weight was classified as normal, over- or underweight according to the criteria of Kromeyer-Hauschild [37].

Consumption of vegetables and fruit was measured among children aged 12 months and older with three questions ‘how often do you/does your child eat: 1) fresh fruit, 2) cooked or 3) uncooked vegetables?’ Possible answer choices for each question were based on a 10-point scale ranging from ‘1‘ (never) to ‘10’ (more than 5 times a day) [38]. The total score which could be reached by the three questions ranged thus from a minimum of 3 to a maximum of 30 points. According to their total score participants were then classified into ‘low’ , ‘intermediate’ or ‘high’ vegetables/fruit consumption groups.

Information on sports activities was collected among children 3–17 years only, which was provided by parents of 3–10 year-old children in the parental self-filled questionnaires and by 11–17 year-old adolescents in the child self-filled questionnaires [39].

### -Parental social status

Parental social status was defined as lower, intermediate or upper according to the total score of a composite social status index integrating the parents’ level of education (primary, middle, higher and other), household income (<1500 €, 1500 - <2250 €, 2250 - <3000 €, and > =3000 €) and profession [40].

### -Parents-related health behavior

Children’s exposure to passive smoking at home was defined by the question ‘Is there any smoking at home in the presence of your child?’ , the frequency of which was categorized as ‘daily’ , ‘sometimes’ and ‘never’.

Data on children’s breastfeeding were collected based on two questions. First, we asked ‘Had your child ever been breastfed?’ If the answer was ‘yes’ , then we asked ‘How long had your child been breastfed *exclusively*, that means no extra bottle feeding or complementary feeding? Answer choices for this question were ‘never breastfed exclusively’ , ‘breastfed exclusively up to XX months’ (number of months should be given) or ‘do not know’ [41].

### Statistical analysis

IBM SPSS Statistics (version 20, SPSS Inc. Chicago, IL) was used for statistical analyses. A weighting factor was used to adjust for deviations of demographic characteristics between the survey population and official population statistics (as of 31th December 2004) [30]. This was a necessary step in order to avoid selection bias caused by the two-stage sampling procedure. Descriptive statistics were used to examine characteristics of the study population and prevalence of herbal medicinal product use. The second-order Rao-Scott chi-square test was used to test for group differences within specific subgroups. We fitted two basic logistic regression models with ‘herbal medicinal product use’ as the dependent variable: Model 1 looking at herbal medicinal product users vs. all children who did not use herbal medicinal products, model 2 looking at herbal medicinal product users vs. users of other medicines except for herbal medicinal products. Independent variables in model 1 and model 2 were: sex, age groups, region 1 (East vs. West Germany), region 2 (North vs. Central vs. South Germany), urbanicity (rural areas vs. small vs. medium sized vs. large cities), BMI, Children with Special Health Care Needs (according to CSHCN-screener), general health status, parental social status and immigration background. Odds ratios (OR) and 95% confidence intervals (95% CI) as a measure of determinants of herbal medicinal product use were obtained from the basic logistic regression models. Children’s and parents-related health behavior variables (sports activities, consumption of fruit and vegetables, exposure to passive smoking, exclusive breastfeeding) were then added into the basic models - one by one separately - to test for independent effects. Because parents’ educational levels and household incomes were components of social status, their independent effects were tested in the basic models with exclusion of the variable of social status. The large sample size of this study allows the exclusion of missing values in multivariable regression modeling without influencing the results. The SPSS Complex Samples method was used in the statistical analysis to account for clustering due to the two-stage sampling procedure. P-values less than 0.05 and/or 95% CIs that did not overlap were considered as statistically significant.

## Results

### Prevalence of herbal medicinal product use

**Figure 1:**
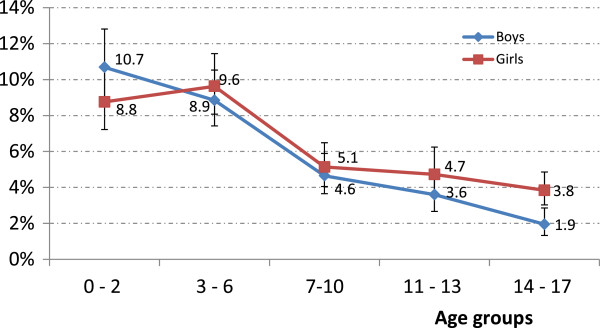
**Prevalence of herbal medicinal product use by age group and sex.** Data source: German National Health Interview and Examination Survey for Children and Adolescents (KiGGS) 2003–2006.

Among 17,450 study subjects, a total of 1,055 children use at least one herbal medicinal product (Table 1). The overall weighted user prevalence is 5.8% (95% CI 5.3-6.3%) with no significant difference (p = .065) between boys (5.5%, 95% CI 4.9-6.1%) and girls (6.2%, 95% CI 5.5-6.9%). Children younger than 6 years old show a higher use of herbal medicinal products compared to children in other age groups. With increasing age, use of herbal medicinal products declines both in boys and girls. Significant differences are found between younger (≤6 years) and older (7–17 years) children. A significant difference between boys and girls is found in the age group 14–17 years only, with girls showing a higher use than boys (3.8% vs. 1.9%, p < .001) (Figure 1). No difference is found among children residing either in East vs. West Germany (p = .287), or in North vs. Central vs. South Germany (p = .181) or in rural areas vs. cities (p = .211) (data not shown).

**Table 1:** Characteristics of study subjects and prevalence of herbal medicinal product use – by health related indicators

		**Study subjects**		**Herbal medicinal product users**										
		N*	%**	n*	%**	**95 %CI**	P***							
***Total***		**17,450**	**100**	**1,055**	**5.8**	**5.3-6.3**								
***Children's health status***							
**Parents-rated health status**							
	*Excellent*	6,873	39.1	366	5.2	4.6-5.9	**.002**
	*Good*	9,250	54.1	592	6.1	5.5-6.8	
	*Fair/bad/very bad*	1,102	6.7	92	7.9	6.2-10.0	
CSHCN-Screener****							
	*Yes*	2,205	13.7	135	6.2	5.1-7.6	.629
	*No*	13,752	86.3	857	5.9	5.4-6.5	
**Body mass index (BMI)**							
	*Underweight*	1,121	7.0	80	6.3	4.8-8.2	.136
	*Normal*	13,567	78.4	833	5.9	5.4-6.5	
	*Overweight*	2,517	14.6	130	4.8	3.8-6.0	
***Children's health behavior***							
**Sports activities**							
**(children aged 3–17 years only)**							
	*Daily*	2,335	16.7	89	3.7	3.0-4.7	**.000**
	*3-5 times/week*	3,119	23.3	142	4.6	3.9-5.6	
	*1-2 times/week*	5,811	40.2	353	6.0	5.2-6.8	
	*1-2 times/month*	1,306	8.5	100	7.3	5.9-9.2	
	*Never*	1,691	11.3	82	4.6	3.6-5.9	
**Vegetable & fruit consumption**							
**(children aged 1–17 years only)**							
	*Low*	3,866	26.6	185	4.8	4.0-5.7	**.002**
	*Middle*	7,415	47.2	455	5.9	5.2-6.7	
	*High*	4,176	26.2	288	7.0	6.1-8.0	
***Parental socio-economic status***							
**Immigration background**							
	*No*	14,790	82.8	957	6.3	5.7-6.9	**.000**
	*Yes*	2,580	17.2	93	3.5	2.8-4.2	
**Maternal education level**							
	*Primary*	3,849	25.4	171	4.3	3.6-5.2	**.000**
	*Middle*	7,836	40.3	510	6.4	5.6-7.2	
	*Higher*	4,838	28.5	339	6.7	6.0-7.6	
	*Others*	927	5.7	35	3.5	2.3-5.3	
**Paternal educationlevel**							
	*Primary*	4,832	31.7	233	4.7	4.0-5.5	**.000**
	*Middle*	5,933	28.1	401	6.5	5.6-7.5	
	*Higher*	5,100	31.3	368	7.0	6.2-7.9	
	*Others*	1,585	9.0	53	3.4	2.5-4.7	
**Household income per month**							
	*<1500 €*	3,558	19.0	183	5.0	4.2-6.0	**.021**
	*1500 - <2250 €*	4,469	26.6	262	5.3	4.5-6.2	
	*2250 - <3000 €*	4,354	27.2	284	6.2	5.4-7.1	
	*> = 3000 €*	3,986	27.2	267	6.6	5.8-7.5	
**Social status**							
	*Lower*	4,760	27.5	234	4.7	4.0-5.5	**.001**
	*Intermediate*	7,901	45.4	506	6.1	5.4-6.9	
	*Upper*	4,366	27.0	307	6.9	6.0-7.8	
***Parents-related health behavior***							
**Exclusive breastfeeding of children**							
	*Never*	3,646	22.6	193	5.1	4.3-6.1	**.010**
	*Combined with formula milk/unclear*	2,493	7.8	130	5.1	4.1-6.3	
	*< 4 months*	3,739	20.8	229	5.6	4.8-6.6	
	*4-5 months*	3,174	18.5	216	6.5	5.6-7.5	
	*≥ 6 months*	3,796	23.5	270	7.0	6.1-8.0	
**Children exposed to passive smoking at home**							
	*Daily*	2,612	16.6	102	3.8	3.1-4.8	**.000**
	*Sometimes*	2,236	13.1	121	5.1	4.1-6.2	
	*Never*	12,137	70.4	820	6.5	5.9-7.2	

*Absolute numbers, unweighted, the sum of subgroups may not be equal to the total because of missing values.

**Percentages, weighted.

***P-values: derived from Rao-Scott chi-square tests for the difference of prevalence estimates within specific groups.

****Children with Special Health Care Needs.

Data source: German Health Interview and Examination Survey for Children and Adolescents (KiGGS) 2003–2006.

Stratified by children’s health status and health behavior as well as parental socio-economic status and parents-related health behavior, a significantly higher herbal medicinal product use is seen among children with a poor (fair/bad/very bad) general health status. This also applies to children engaging moderately (1–2 times/month and 1–2 times/week) in sports activities, children with a high vegetable and fruit consumption, children who had been exclusively breastfed ≥6 months and children who had never been exposed to passive smoking at home. Under the same stratification we also find a significantly higher herbal medicinal product use among children with no immigration background, children whose father and mother have a higher level of education, household income and social status. No association is found with regard to children’s BMI and between children with and without special health care needs (Table 1).

### Patterns of herbal medicinal product use

**Figure 2:**
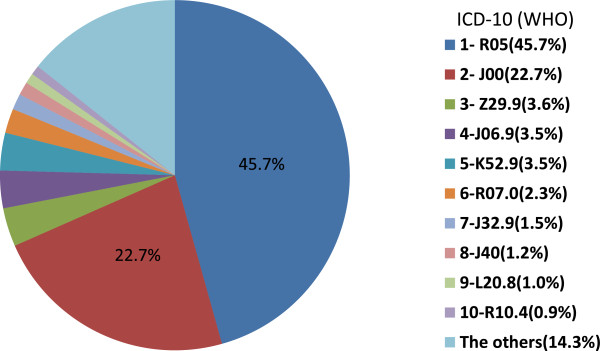
**Indications of herbal medicinal products (N = 1,152).** Data source: German National Health Interview and Examination Survey for Children and Adolescents (KiGGS) 2003–2006. ICD-10, WHO: R05: Cough; J00: Acute nasopharyngitis (common cold); Z29.9: Prophylactic measure; J06.9: Acute upper respiratory infection; K52.9: Noninfective gastroenteritis and colitis; R07.0: Pain in throat and chest; J32.9: Chronic sinusitis; J40: Bronchitis, not specified as acute or chronic; L20.8: other atopic dermatitis; R10.4: other and unspecified abdominal pain.

Of 17,450 study participants 8,899 are users of medicine. They utilized a total of 14,588 preparations within the last 7 days. 1,152 (7.9%) of those preparations are herbal medicinal products. More than two thirds (71.9%) of all herbal medicinal products are used for the treatment of cough, common cold and acute upper respiratory infection (in the following referred to as “coughs and colds”). The other indications of herbal medicinal products are less frequently mentioned, all less than 5%. Notably, 3.6% of all herbal medicinal products are used as a prophylactic measure (Figure 2).

Overall 44.5% of herbal medicinal products are prescribed by medical doctors, which is a significantly lower prescription rate compared to conventional medicinal products (67.7%). Herbal medicinal products are more likely to be used for a shorter term (<1 week) in comparison with conventional medicinal products (77.0% vs. 49.8%). In terms of improvement of conditions treated, compared to conventional medicines, more herbal medicinal products are rated as ‘partly’ (42.4% vs. 35.3%) and less herbal medicinal products are rated as ‘greatly’ effective (46.8% vs. 55.9%) (Table 2). When combining the categories ‘greatly’ and ’partly’ together, herbal medicinal products are less likely to improve the conditions treated than conventional medicinal products (89.2%, vs. 91.2%, p = .042, data not shown in Table 2). However, 3.3% of herbal medicinal products show no effect at all, which does not significantly differ from conventional medicinal products (2.6%). Overall, 10 adverse drug events are recorded among herbal medicinal products with a proportion of 0.9% compared with 125 adverse drug events (1.2%) recorded among conventional medicinal products (p >0.05, data not shown).

**Table 2:** Patterns of herbal compared to conventional medicinal product use

	***Herbal medicinal products***			***Conventional medicinal products***			P*
	**n**	**%**	**95% CIs**	**n**	**%**	**95% CIs**	
**Total number of drugs**	**1,152**	**100**	**-**	**10,433**	**100**	**-**	
***Origin***							
Prescribed by doctors	510	44.5	41.65-47.39	7012	67.7	66.8-68.6	.000
Bought OTC	425	37.1	34.34-39.92	2077	20.1	19.3-20.8	
Obtained from other sources	211	18.4	16.28-20.77	1264	12.2	11.6-12.9	
*Missing*	*6*			*80*			
***Duration of use***							
<1 week	882	77.0	74.4-79.3	5114	49.8	48.8-50.8	.000
1-4 weeks	221	19.3	17.1-21.7	1409	13.7	13.1-14.4	
1-12 months	25	2.2	1.5-3.2	2022	19.7	18.9-20.5	
1 year or longer	18	1.6	1.0-2.5	1724	16.8	16.1-17.5	
*Missing*	*6*			*164*			
***Self-rated improvement of conditions treated***							
Greatly	505	46.8	43.9-49.8	3841	55.9	54.7-57.0	.000
Partly	457	42.4	39.5-45.4	2426	35.3	34.1-36.4	
Not much	80	7.4	6.0-9.2	427	6.2	5.7-6.8	
Not at all	36	3.3	2.4-4.6	181	2.6	2.3-3.0	
Doesn’t apply**	*39*			*3199*			
*Missing*	*35*			*359*			
***Tolerance of drug use***							
Very good/good	1,130	98.9	98.1-99.4	10182	98.4	98.2-98.6	.163
Partly/not tolerated	12	1.1	0.6-1.9	164	1.6	1.4-1.8	
*Missing*	*10*			*87*			

*P-value: chi-square test for differences in proportions between herbal and conventional medicinal products.

**Drugs were used e. g. as a preventive measure, for contraception, or diagnosis was unknown.

Data source: German Health Interview and Examination Survey for Children and Adolescents (KiGGS) 2003–2006.

Of 1,152 herbal medicinal products, 711 (61.7%) are mono-products containing one herbal active substance. 231 (20.1%) are combination products with two herbal active substances while 210 (18.2%) are combination products with multiple herbal active substances (range 3–9). Ivy leaves are used most frequently, found in one third (32.2%) of all herbal medicinal products and in 43.9% of mono-herbal medicinal products (Table 3). Among combination products with two herbal active substances, eucalyptus leaves with pine needles (31.2%) is the most frequently reported combination, followed by the combinations of thyme herbs with ivy leaves (24.7%), thyme herbs with primrose roots (22.5%) and eucalyptus leaves with spruce-needles (9.5%) (data not shown).

**Table 3:** The 10 most frequently used herbal active substances (unprocessed herbal substances or herbal preparations) in mono- and in all herbal medicinal products

**Herbal medicinal mono-products (n = 711)**	**n (%)**	All herbal medicinal products (N = 1152*)	**n (%)**
Ivy leaf	312(43.9)	Ivy leaf	371(32.2)
Pelargonium root	112(15.8)	Thyme herb	217(18.8)
Thyme herb	71(10.0)	Eucalyptus leaf	133(11.5)
Saccharomyces boulardii	37(5.2)	Pelargonium root	111(9.6)
Purple coneflower herb	22(3.1)	Elder flower	77(6.7)
Ribwort plantain herb	15(2.1)	Primula flower	77(6.7)
Evening primrose seed	11(1.5)	Verbena herb	77(6.7)
Iceland moss	10(1.4)	Gentian root	77(6.7)
Fennel fruit	9(1.3)	Sorrel herb	77(6.7)
Matricaria flower	7(1.0)	Matricaria flower	75(6.5)

Data source: German Health Interview and Examination Survey for Children and Adolescents (KiGGS) 2003–2006.

### Determinants of herbal medicinal product use

A multivariable regression model with herbal medicinal product users vs. all children who did not use herbal medicinal products (model 1) shows the following results: Herbal medicinal product use among the pediatric population is significantly and positively associated with younger age, residing in the south of Germany, having no immigration background, having a poor general health status and coming from families with higher socio-economic status (Table 4, model 1). Comparable determinants with exception of a positive CSHCN-screener are also found in model 2 with herbal medicinal product users vs. users of other medicines (except herbal medicinal products) as the dependent variable. Children with special health care needs are less likely to use herbal medicinal products (Table 4, model 2). No association is found for sex, region 1 (East vs. West), urbanicity (rural areas vs. small vs. medium sized vs. large cities) and BMI in both model 1 and model 2 (all p > .05, data not shown).

**Table 4:** Determinants of herbal medicinal product use

	Model 1*, R^**2**^ = **.06**		Model 2**, R^**2**^ **= .06**	
	**(Total study population N = 17,450)**		**(Medicine users only n = 8,899)**	
	**Adjusted OR**	**95% CI**	**Adjusted OR**	**95% CI**
**Age group in years**				
0 – 2	4.21	3.19-5.56	2.72	2.04-3.62
3 – 6	3.71	2.82-4.88	3.80	2.88-5.02
7 – 10	1.84	1.37-2.47	2.27	1.68-3.07
11 – 13	1.61	1.20-2.15	2.02	1.50-2.73
14 – 17	1			
**Region 2**				
Northern	1		1	
Central	1.23	0.97-1.55	1.27	1.02-1.58
Southern	1.37	1.05-1.79	1.32	1.02-1.70
**Immigration background**				
No	1.65	1.28-2.11	1.36	1.06-1.76
Yes	1		1	
**General health status**				
Excellent	1		1	
Good	1.48	1.28-1.70	1.31	1.13-1.53
Fair/bad/very bad	2.17	1.59-2.98	1.67	1.20-2.32
CSHCN-screener***				
Yes	1.05	0.83-1.34	0.71	0.56-0.91
No	1		1	
**Social status**				
Lower	1		1	
Intermediate	1.32	1.10-1.60	1.28	1.05-1.56
Higher	1.41	1.14-1.73	1.29	1.04-1.60

OR = odds ratio, 95% CI = 95% confidence interval.

*Herbal medicinal product users vs. those who do not use herbal medicinal product as the dependent variable.

**Herbal medicinal product users vs. users of other medicine (except for herbal medicinal products) as the dependent variable.

***Children with Special Health Care Needs.

Other variables included in model 1 and model 2: sex, region 1 (East vs. West), urbanicity (rural areas vs. cities) and BMI.

Data source: German Health Interview and Examination Survey for Children and Adolescents (KiGGS) 2003–2006.

Adding children’s and parents-related health behavior variables including sports activities, consumption of fruit and vegetables, exposure to passive smoking at home and exclusive breastfeeding - one by one independently - into the two basic models, no associations with usage of herbal medicinal products are found to be statistically significant (data not shown).

Replacing social status with maternal and paternal education levels or household income - one by one independently - in the models, we found that both maternal and paternal education levels, but not household income, are statistically significant (data not shown).

## Discussion

### Principle findings

In a national representative sample of children and adolescents aged 0–17 years in Germany, 5.8 % of study participants utilized herbal medicinal products within the last 7 days. Use of herbal medicinal products declines along with increasing age and shows no difference between boys and girls in younger age groups. Teenage (14–17 years) girls are more likely to use herbal medicinal products than teenage boys. Two thirds of herbal medicinal products are used for the treatment of coughs and colds; nearly half of herbal medicinal products are prescribed by medical doctors. Use of herbal medicinal products is closely associated with younger age, residing in the south of Germany, having a poor health status, having no immigration background and coming from a higher social class family. Children’s and parents-related health behavior is not found to be associated with herbal medicinal product use after adjusting for social class.

### Prevalence of herbal medicinal product use

Many factors can influence the prevalence rates, which vary considerably between studies depending on study methodology. Any comparisons between studies should consider differences in study population, observational window, data collection mode, definition of herbal medicinal products etc. Most international studies are conducted among children with a specific chronic condition [16,18] or among inpatients and outpatients [20-22] and show a higher herbal medicinal product use. Longer observational windows cover more herbal medicinal product use and thus account for higher prevalence rates.

The prevalence estimate of herbal medicinal product use found in our study is higher than rates reported in a study with a similar setting from the USA. The Slone Survey, a telephone survey of medicine use in the US population, collected data on the use of prescription and non-prescription medication including herbal products/natural supplements during the previous week [25]. Results of this survey report a rather low use of herbal medicine in the past 7 days at < 0.5% among children 0–12 years [25]. In comparison, our 7-days prevalence amounts to 5.8% among children aged 0–17. The rather low herbal medicine use among US children is confirmed by the results of the 2007 National Health Interview Survey, in which the 12-month prevalence rate of natural products usage (mostly herbal medicines) among children aged 0–17 years amounts to 3.9% [26]. Even among US-children presented for surgery, only 3.5% of the patients had been given herbal or homoeopathic medications in the preceding 2 weeks [42].

In a population-representative sample of approximately 6,000 Italian families, unconventional medicinal product use is investigated in the frame of a study on ‘Health conditions and health service utilization’ [24,43]. Prevalence of herbal medicine use among children aged 0–13 years during the 3-years period between 1997 and 1999 amounts to 2.4% [24]. Similarly, an investigation in South Australia including 911 children aged 15 years or less finds that 168 children (18.4%) use CAM in the previous 12 months. One third of these CAM users (n = 56) are herbal medicine users [44], which equals a 12-month prevalence rate of 6.1%. An investigation into the use of Traditional Chinese Medicine (TCM), an Asian medicine system consisting mainly of herbal medicine therapy, among 5,971 Chinese children in Taiwan finds that 4.7% of the participants used TCM within the past month [45]. Even though the prevalence rates of the South-Australian [44] and Taiwanese [45] studies come close to our results, it is difficult to compare rates as the observation periods vary greatly.

Herbal medicine and homoeopathy are the two frequently used CAMs in German-speaking countries [15,27,28,46]. Based on KiGGS data, we previously reported a pediatric homoeopathic medicinal product use of 4.6% [47] in Germany, slightly lower than herbal medicinal product use in the present study (5.8%). Another German study including two birth cohorts of a total of 3,642 children shows a 4-weeks prevalence rate of herbal medicinal product and homoeopathic medicinal product use of 8.9% and 14.3%, respectively [15], both higher than KiGGS results. Yet, methodologically, this study is not comparable to KiGGS, as they look at a different age group (9.4 to11.6 vs. 0 to 17 years) and the observation period differs from KiGGS (4 weeks vs. 7 days) [15]. Additionally, the proportion of children’s parents with higher education and income levels is overrepresented compared to German national means [15], which may contribute to a higher use of herbal and homoeopathic medicinal products.

Possible reasons for a high herbal medicinal product use in our study compared to international analyses are various. In Germany, the treatment of children under 12 years and of children with developmental disorders up to 18 years, with herbal or other alternative medicine is reimbursed by the health insurance, even if the medication is available without a prescription (over the counter, OTC). Further, OTC herbal medicinal products can be recommended or prescribed by non-medical practitioners called ‘*Heilpraktiker*’ in Germany. A *Heilpratiker* receives no university medical school training but obtains a state license after passing a specific examination usually after completion of an optional medical knowledge course [48]. The number of non-medical practitioners *Heilpraktiker* has increased recently in Germany [49,50], possibly playing a role in the increase of herbal medicinal product use. Nevertheless, in the comparison between studies, we should also bear in mind the differences in the definition of herbal medicinal product, which may lead to different prevalence rates. Differences in cultural and traditional background as well as differences in access to health care systems can influence the prevalence rates of studies in different countries.

### Patterns of herbal medicinal product use

Similar to our study, previous studies conducted in Germany investigating the patterns of herbal medicinal product use also find that the vast majority of herbal medicinal products are used to treat coughs and colds, followed by herbal treatments for intestinal disorders [15,20]. The predominance of the indications which can be summarized as coughs and colds is in line with the fact, that most of newly licensed herbal medicinal products belong to this therapeutic area [51]. In the present study almost half of all herbal medicinal products are prescribed by medical doctors, which is significantly less compared to that of conventional medicinal products, but significantly more compared to homoeopathic medicinal products (about 25%) [47]. Concerning the effectiveness of treatment, we find that nearly half (46.8%) of all herbal medicinal products can improve the condition treated ‘greatly’ , which is significantly lower than that of conventional medicinal products (55.9%). A different pattern occurs when looking at the answer category ’condition improved partly’; significantly more herbal medicinal products than conventional medicinal products were rated to be ‘partly’ effective. Overall, only a very small part (3.3%) of herbal medicinal products is rated to be not effective at all, which shows no difference from that of conventional medicinal products. However, this does not allow any conclusion on effectiveness when comparing herbal and conventional medicinal products, as there are differences in the conditions treated by the two types of medicines. Two thirds of herbal medicinal products are used for the treatment of coughs and colds, which in most cases are self-limiting diseases in children. In contrast, conventional medicinal products are used for the treatment of a wide range of acute and chronic conditions.

### Determinants of herbal medicinal product use

Determinants of herbal medicinal product use in the present study include children residing in South Germany, and coming from families with higher socio-economic status, mainly educational level. This is well in line with results of a previous regional population-based study conducted in Germany [15]. This study finds that herbal medicinal product use is significantly positively associated with a higher maternal educational level, but reversely associated with living in West (Wesel-area) compared with South Germany (Munich) [15]. Children of families with higher socio-economic status are found to have a significantly higher use of CAM [52] including herbal medicines [45]. Like in our study, household income is not found to be associated with herbal medicinal product use [15]. The influence of social status can be explained by maternal and paternal education levels, but not by household income in our study. Possibly, this may be due to the fact that herbal medicinal products prescribed for children less than 12 years are reimbursed by health insurances in Germany. Children with immigration background in Germany are associated with a lower parental socio-economic status [35], this might be one of the reasons why they are less likely to use herbal medicinal products. Other possible reasons could be rooted in cultural and religious differences among children with immigration background. Previous studies have shown that in German-speaking countries homoeopathic medicine use is more common in families with no immigration background [47]. Yet, an American study [53] found no difference in herbal medicine use according to race and country of origin of the child. Another American study looking at low-income, nutritionally vulnerable children found that the use of herbals is more common among Latino children compared to all other children of the study, but does not differ among the two states in which the studies were conducted (Wisconsin and Kansas) [22]. Younger children < 6 years are the main herbal medicinal product users in our study. This may reflect in part the broad acceptance of herbal medicinal products based on the assumption that herbal medicine is ‘mild’ and associated with less undesirable effects than allopathic medicine [54]. This popular attitude may be decisive especially for the treatment of young children who are considered to be the most vulnerable group.

In our study the CSHCN-screener is found not to be associated with herbal medicinal product use in the logistic regression model 1 while inversely associated with herbal medicinal product use in model 2. This implies that children with special health care needs are more likely to be treated with conventional medicinal products rather than herbal medicinal products.

### Strengths and limitations

One of the strengths of our study can be seen in the inclusion of a large population-representative community sample of a national health survey. Further, we asked survey participants/children’s parents to bring the original packages and/or inserts to the examination sites for the purpose of verification of drug use, and we investigated medicine use for a short period of 7 days prior to the interview, both contributing to reduce recall bias. Classification of herbal medicinal products in the present study was confirmed independently by health care professionals and regulatory experts of federal pharmaceutical supervising authorities. In this way, misclassification bias is reduced to the least degree.

However, there are several limitations to our study. First, our study subjects are children living in communities; children with severe medical diseases requiring inpatient treatment are not included in our study. These children may have a different pattern of medication use, including herbal medicinal products. Second, subject to cross-sectional design, our study does not allow to draw any causality conclusions. Third, though we cover as many influence factors as possible in the regression models, other factors that may have a substantial influence on children’s health are not considered. So could for example, the severity of a disease influence the use of alternative medicine [55].

## Conclusions

In summary, we found a relatively high use of herbal medicinal products among children in Germany in comparison with children from other countries. Herbal medicinal products are mainly used for the treatment of coughs and colds among children. A high proportion of herbal medicinal products is prescribed by medical doctors, suggesting a certain degree of acceptance among medical doctors in Germany. Determinants of herbal medicinal product use include younger age, residing in South Germany, having a poor health status, having no immigration background and a higher parental socio-economic status, mainly educational level. Children's and parents-related health behaviors are found not to be associated with herbal medicinal product use after adjusting for social class. Findings of this study provide essential data and a reference on the use of herbal medicinal products for policy-makers, health professionals and parents. Further studies are needed to investigate the effectiveness and safety of specific herbal medicinal products, potential effects of long term use as well as possible interactions of herbal medicines with concomitantly used conventional medicinal products.

## Competing interests

The authors declare that they have no competing interests.

## Authors’ contributions

YD performed the statistical analysis, wrote and finalized the manuscript. IW assisted in analyzing the data and interpreting the results, writing and finalizing the manuscript. WZ conducted the data classification, and assisted in analyzing the data and interpreting the results. SB conducted the reclassification of the data, the quality control and reviewed the manuscript. WK provided specific knowledge, assisted in the conceptualization of the study, and contributed to the manuscript. HK coordinated the conceptualization and conduction of the project. HK is the guarantor for the study. All authors read and approved the final manuscript.

## Pre-publication history

The pre-publication history for this paper can be accessed here:

http://www.biomedcentral.com/1472-6882/14/218/prepub
